# Supplementary material for: Microglia govern the extinction of acute stress-induced anxiety-like behaviors in male mice
Source: Nat Commun. 2024 Jan 10;15:449. doi: 10.1038/s41467-024-44704-6 (PMC10781988; doi:10.1038/s41467-024-44704-6)
Supplement: Supplementary file 1 — Supplementary Information [file 41467_2024_44704_MOESM1_ESM.pdf]

Title: Microglia govern the extinction of acute stress-induced anxiety-like behaviors in male mice

Authors: Danyang Chen[1†], Qianqian Lou[1†], Xiang-Jie Song[1†], Fang Kang[1], An Liu[2], Changjian Zheng[3], Yanhua Li[1], Di Wang[1], Sen Qun[4], Zhi Zhang[1, 5]*, Peng Cao[4]*, Yan Jin[4]*

Supplemental figure titles and legends

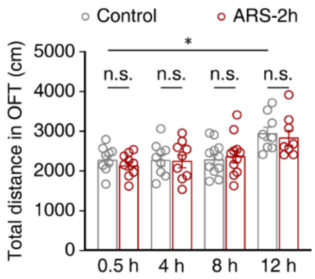

Supplementary Fig. 1: Performance of ARS-treated mice in open field test at different time point.

Summarized data of movement distances in central area of OFT in ARS-2h mice at 0.5 h, 4 h, 8 h, and 12 h post-stress induction and corresponding control mice. Different batches of mice were used for each OFT assay (0.5 h, n = 9 mice per group; 4 h, n = 9 mice per group; 8 h, n = 11 mice per group; 12 h, n = 8 mice per group; $F_{1,66} = 0.2290$, $p = 0.6338$). Significance was assessed by two-way repeated-measures ANOVA with post hoc comparison between groups. All data are presented as mean ± SEM. *$p < 0.05$; n.s., not significant. See also Supplementary Data 1. Source data are provided as Source Data file.

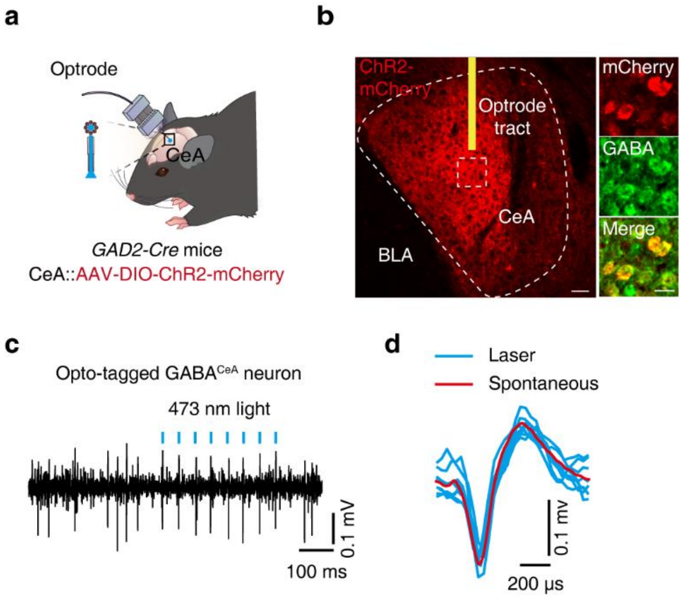

Supplementary Fig. 2: Identification of characteristic GABA^{CeA} neuron spike waveforms. **a**, Schematic for optogenetic tagging and electrophysiological recording. Enlarged area shows optrodes. **b**, Representative images of virus injection site in the CeA (left) and mCherry^{+} neurons colocalized with immunofluorescence signal for GABAergic neurons (right). Scale bars, 50 µm (left) and 20 µm (right). **c**, **d**, Example recording of spontaneous and light-evoked spikes from a GABA^{CeA} neuron (**c**) and overlay of averaged spontaneous (red) and light-evoked (blue) spike waveforms from the example unit (**d**).

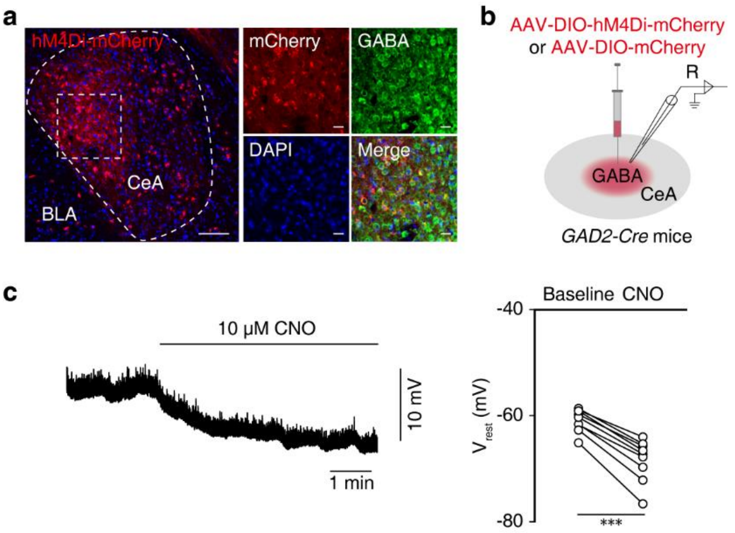

Supplementary Fig. 3: Chemogenetic inhibition virus successfully expresses in $GABA^{CeA}$ neurons.

a, Representative images of CeA-injection sites (left) and mCherry-labeled neurons (red) co-labeled with GABA immunofluorescence (right). Scale bars, 100 μm (left) and 20 μm (right). **b**, Schematic of CeA injection of AAV-DIO-hM4Di-mCherry in *GAD2-Cre* mice and recording configuration in acute slices. **c**, Whole-cell recordings showing the effect of CNO on AAV-DIO-hM4Di-mCherry expressing $GABA^{CeA}$ neurons (n = 9 cells from three mice per group; $t_8 = 11.23$, $p < 0.001$). Significance was assessed by two- tailed paired Student's *t*-test in (**c**). All data are presented as mean ± SEM. ***$p < 0.001$. See also Supplementary Data 1. Source data are provided as Source Data file.

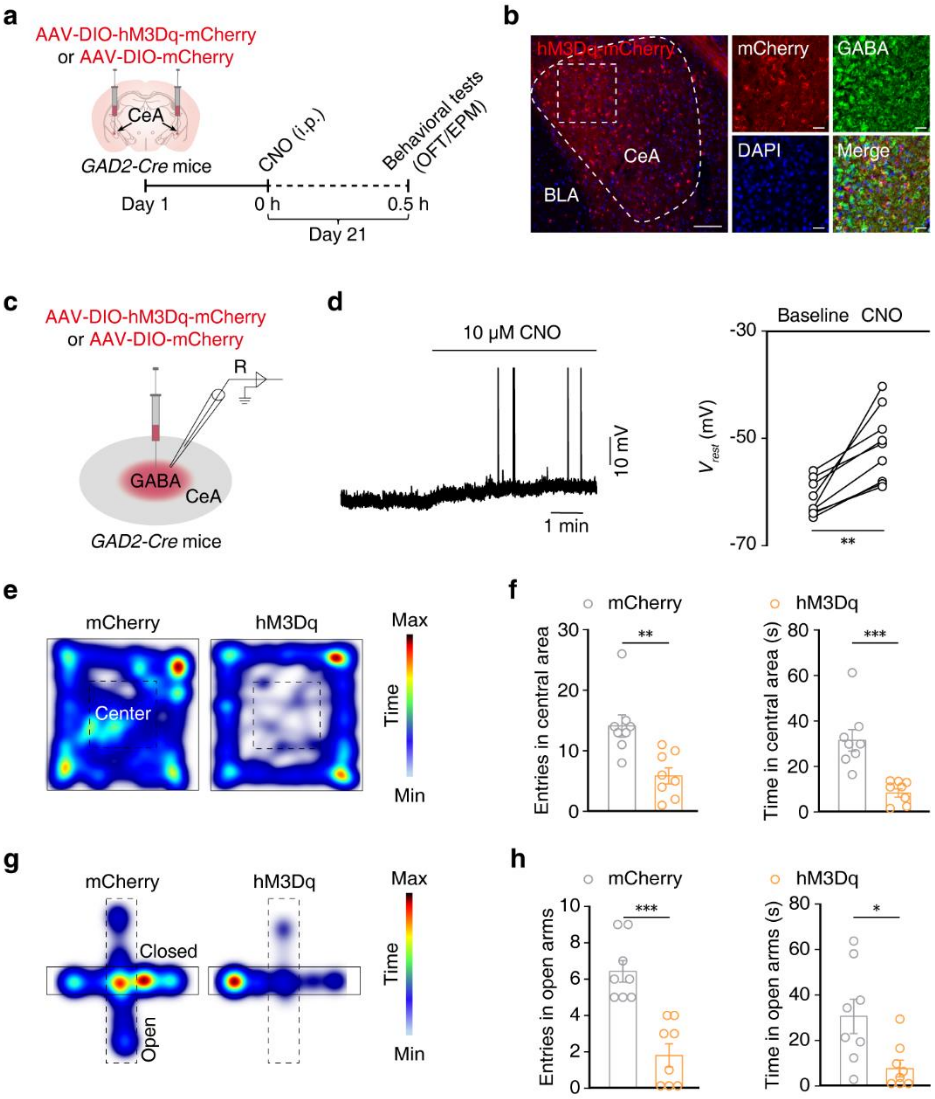

Supplementary Fig. 4: Chemogenetic activation of GABA^{CeA} neurons induces anxiety-like behaviors in na ïve mice.

a, Experimental schematic of chemogenetic activation of GABA^{CeA} neurons and behavioral tests. **b**, Representative images of CeA injection sites (left) and mCherry-labeled neurons (red) co-labeled with GABA immunofluorescence (right). Scale bars, 100 μm (left) and 20 μm (right). **c**, Schematic of CeA injection of AAV-DIO-hM3Dq-mCherry in *GAD2-Cre* mice and recording configuration in acute slices. **d**, Whole-cell recordings showing the effect of CNO on AAV-DIO-hM3Dq-mCherry expressing GABA^{CeA} neurons (n = 9 cells from three mice per group; t_8 = 4.791, p = 0.0014). **e**, **f**, Representative heatmaps of trajectories (**e**) and summarized data of entries and the time spent in central area (**f**; left, t_{14} = 3.641, p = 0.0027; right, t_{14} = 4.559, p = 0.0004) of OFT (n = 8 mice per group). **g**, **h**, Representative heatmaps of trajectories (**g**) and summarized data of entries and the time spent in the open arms (**h**; left, t_{14} = 5.286, p = 0.0001; right, t_{14} = 2.730, p = 0.0163) of EPM (n = 8 mice per group). Significance was assessed by two- tailed paired Student's t-test in (**d**), two-tailed unpaired Student's t-test in (**f, h**). All data

are presented as mean ± SEM. *$p < 0.05$, **$p < 0.01$, ***$p < 0.001$. See also Supplementary Data 1. Source data are provided as Source Data file.

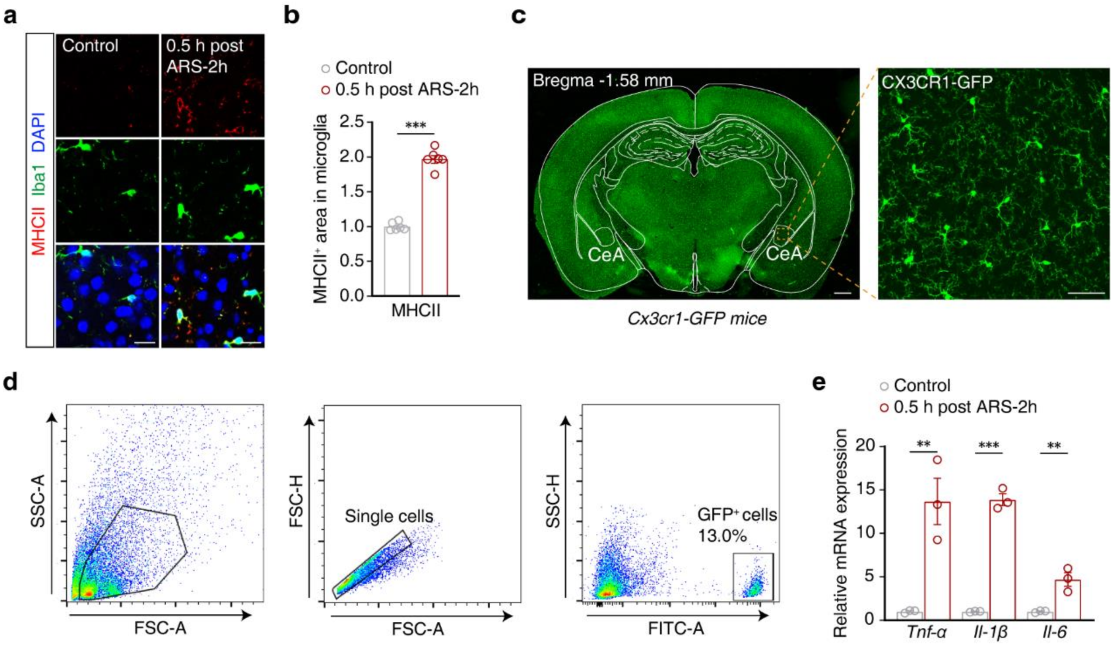

Supplementary Fig. 5: Immunofluorescent staining for the inflammatory molecules in microglia.

a, b, Representative images (**a**) and quantitative analyses (**b**) of immunostaining for MHCII (red), Iba1 (green), and DAPI (blue) in the CeA of 0.5 h post ARS-2h and corresponding control mice (n = 6 mice per group; **b**, t_{10} = 7.769. p < 0.001). Scale bars, 20 μm. **c**, Representative images of microglia in the CeA of *Cx3cr1-GFP* mice. Scale bars, 500 μm (left) and 50 μm (right). **d**, Gating strategy of the cell subpopulations in the CeA GFP^{+} microglia analyzed by flow cytometry. **e**, qPCR analysis of *Tnf-α*, *Il-1β* and *Il-6* mRNA levels in CeA microglia of ARS-2h and control mice (n = 3 samples per group; *Tnf-α*, t_4 = 4.751, p = 0.0090; *Il-1β*, t_4 = 18.98, p <0.001; *Il-6*, t_4 = 4.687, p = 0.0094). Significance was assessed by two-tailed unpaired Student's t-test in (**b, e**). All data are presented as mean ± SEM. **p < 0.01, and ***p < 0.001. See also Supplementary Data 1. Source data are provided as Source Data file.

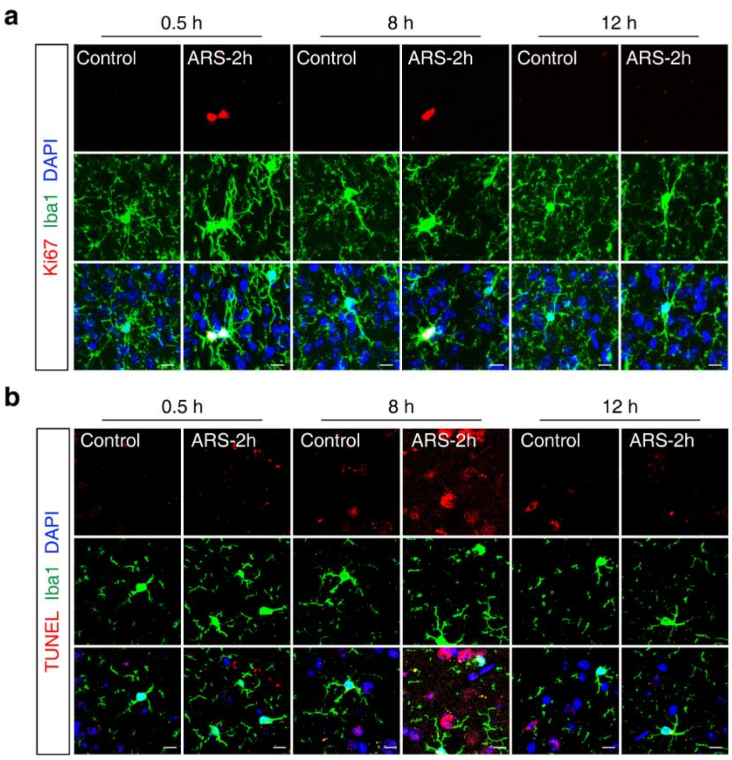

Supplementary Fig. 6: Immunofluorescence staining for Ki67 and TUNEL assays following ARS-2h treatment in mice.

a, Representative images of immunostaining for Ki67 (red), Iba1 (green), and DAPI (blue) in the CeA of ARS-2h and control mice at 0.5 h/8 h/12 h post-stress induction. Scale bars, 10 μm.

b, Representative images TUNEL assays; fragmented DNA (red), Iba1 (green), and DAPI (blue) in the CeA of ARS-2h and control mice at 0.5 h/8 h/12 h post-treatment. Scale bars, 10 μm.

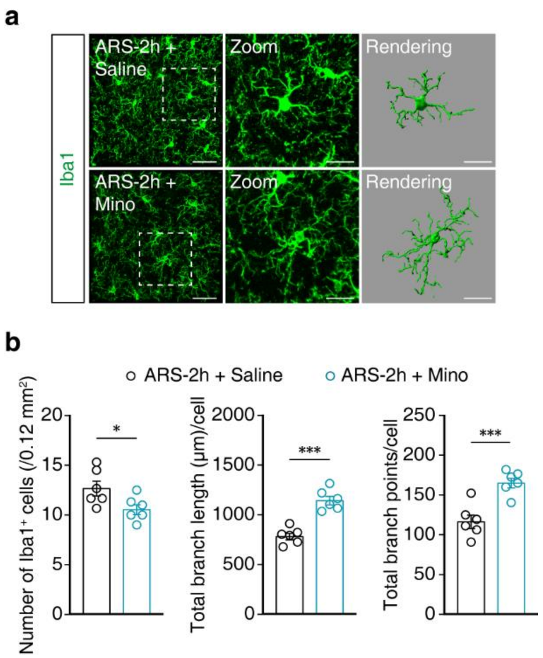

Supplementary Fig. 7: Minocycline inhibits acute stress-induced microglial activation in the CeA.

a, Representative images of Iba1 immunostaining and 3D reconstruction of microglia in the CeA from ARS-2h mice pre-treated with saline or Mino. Scale bars, 40 μm (overview) and 20 μm (inset and rendering). **b**, Quantification of $Iba1^+$ cell numbers, total process length, and number of branch points of microglia in the CeA from ARS-2h mice pre-treated with saline or Mino (n = 6 mice per group; left, $t_{10} = 2.360$, $p = 0.04$; middle, $t_{10} = 6.729$, $p < 0.001$; right, $t_{10} = 4.672$, $p = 0.0009$). Significance was assessed by two-tailed unpaired Student's *t*-test in (**b**). All data are presented as mean ± SEM. $^*p < 0.05$, $^{***}p < 0.001$; n.s., not significant. See also Supplementary Data 1. Source data are provided as Source Data file.

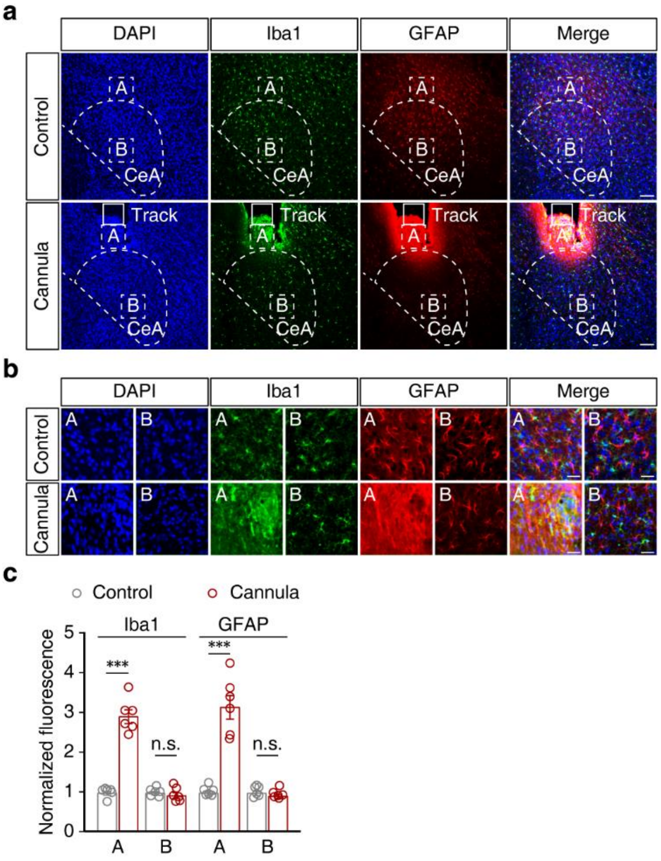

Supplementary Fig. 8: Immunofluorescent staining of gliosis in the CeA and adjacent regions of naïve mice with or without cannular implantation.

a, b, Representative image (**a**) and enlarged image (**b**) of immunostaining for Iba1 and GFAP around the cannula position in the CeA of implanted mice and the same position in control mice. Scale bars, 100 μm (**a**) and 20 μm (**b**). **c**, Quantitative analyses of immunostaining for Iba1 and GFAP in (**b**) (n = 6 mice per group; Iba1, $F_{1,20}$ = 89.37, $p < 0.0001$; GFAP, $F_{1,20}$ = 46.00, $p < 0.001$). Significance was assessed by two-way repeated-measures ANOVA with post hoc comparison between groups in (**c**). All data are presented as mean ± SEM. ***$p < 0.001$; n.s., not significant. See also Supplementary Data 1. Source data are provided as Source Data file.

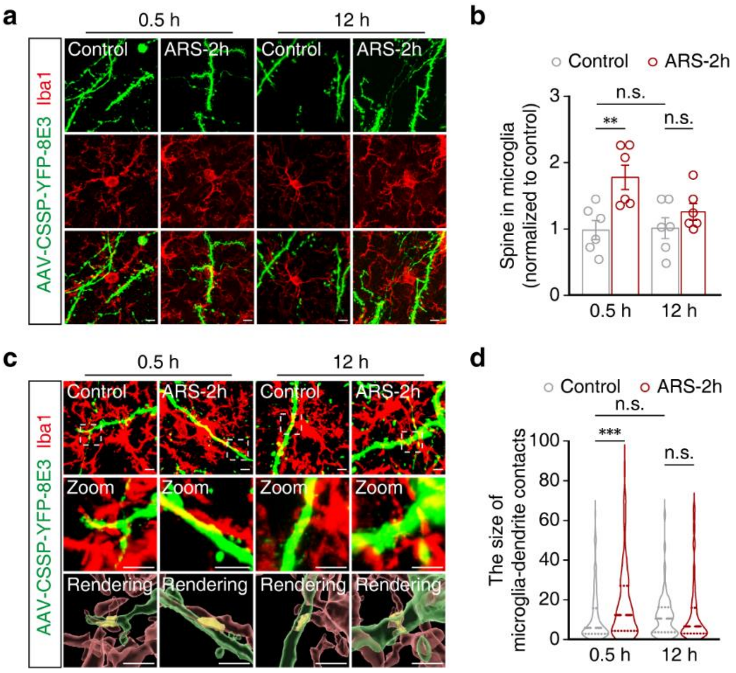

Supplementary Fig. 9: Acute stress increases the size of microglia-dendrite contacts in the CeA.

a, **b**, Reconstructed images (**a**) and summarized data (**b**) for the number of microglia-dendritic spines of Iba1$^+$ microglia (red) containing YFP$^+$ neuronal dendritic spines in the CeA from corresponding control or 0.5 h/12 h post-stress induction mice (n= 6 mice per group; $F_{1,20}$ = 11.76, p = 0.0027). Scale bars, 5 µm. **c**, **d**, Reconstructed images (**c**) and summarized data (**d**) for the size of microglia-dendrite contacts of Iba1$^+$ microglia (red) and YFP$^+$ neuronal dendrites in the CeA from control or 0.5 h/12 h post-stress induction mice (0.5 h control, n = 128 cells from six mice; 0.5 h post ARS-2h, n = 126 cells from six mice; 12 h control, n = 124 cells from six mice; 12 h post ARS-2h, n = 123 cells from six mice; $F_{1,497}$ = 7.912, p = 0.0051). Scale bars, 10 µm (overview) and 5 µm (inset and rendering). Significance was assessed by two-way repeated-measures ANOVA with post hoc comparison between groups in (**b, d**). All data are presented as mean ± SEM. **p < 0.01, ***p < 0.001; n.s., not significant. See also Supplementary Data 1. Source data are provided as Source Data file.

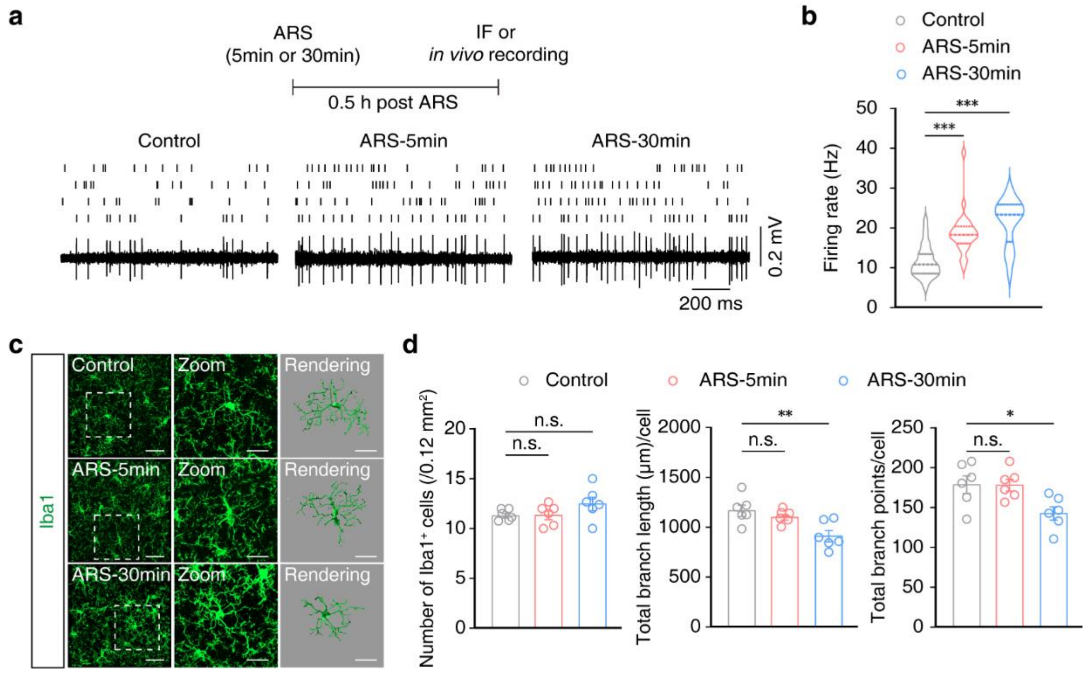

Supplementary Fig. 10: Activation of GABA^{CeA} neurons precedes microglial activation in stress mice.

a, Experimental schematic of ARS-treated mice (top). Raster plots and typical traces (bottom) of the spontaneous firings of GABA^{CeA} neurons in control and ARS-treated mice. **b**, Summary data of firing rate of GABA^{CeA} neurons in control and ARS-treated mice (n = 28 cells from six mice per group; $F_{2,81} = 28.17$, $p < 0.001$). **c**, Representative images of Iba1 immunostaining and 3D reconstruction of microglia in the CeA of control and ARS-treated mice. Scale bars, 40 μm (overview) and 20 μm (inset and rendering). **d**, Quantification of Iba1^{+} cell numbers, total process length, and the number of branch points of microglia in the CeA from control and ARS-treated mice (n = 6 mice per group; left, $F_{2,15} = 2.028$, $p = 0.1662$; middle, $F_{2,15} = 7.389$, $p = 0.0058$; right, $F_{2,15} = 5.137$, $p = 0.02$). Significance was assessed by one-way ANOVA with post hoc comparison between groups in (**b**, **d**). All data are presented as mean ± SEM. *$p < 0.05$, **$p < 0.01$, ***$p < 0.001$; n.s., not significant. See also Supplementary Data 1. Source data are provided as Source Data file.

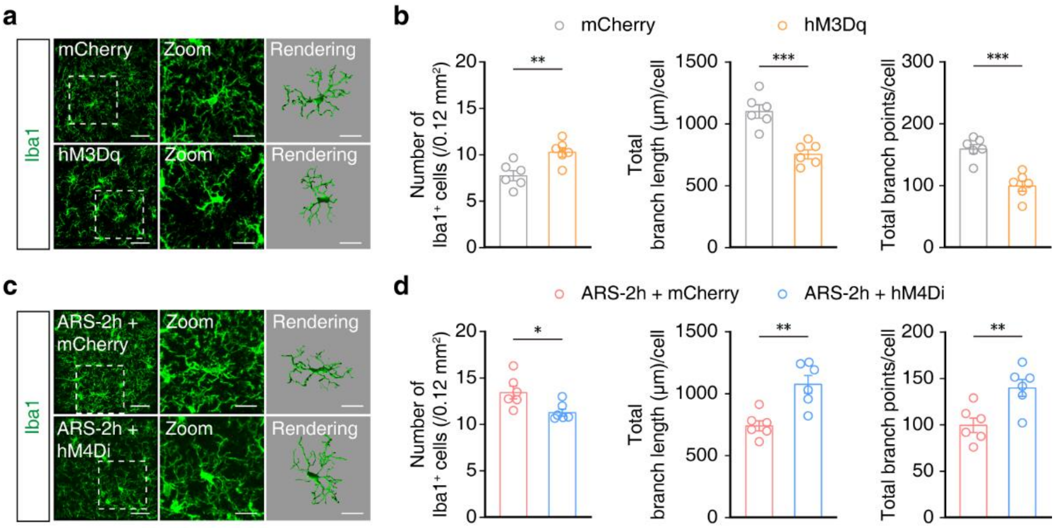

Supplementary Fig. 11: Microglial activation is abolished by chemogenetic inhibition of GABA^{CeA} neurons in acute stress mice.

a, **b**, Representative images of Iba1 immunostaining and 3D reconstruction of microglia (**a**) and quantification of Iba1^{+} cell numbers, total process length, and the number of branch points of microglia (**b**; left, t_{10} = 3.475, p = 0.006; middle, t_{10} = 5.351, p = 0.0003; right, t_{10} = 5.394, p = 0.0003) in the na ïve mice infected with mCherry or hM3Dq-mCherry within the CeA (n = 6 mice per group). Scale bars, 40 μm (overview) and 20 μm (inset and rendering). **c**, **d**, Representative images of Iba1 immunostaining and 3D reconstruction of microglia (**c**) and quantification of Iba1^{+} cell numbers, total process length, and the number of branch points of microglia (**d**; left, t_{10} = 2.754, p = 0.0204; middle, t_{10} = 4.062, p = 0.0023; right, t_{10} = 3.365, p = 0.0072) in the ARS-2h mice infected with mCherry or hM4Di-mCherry within the CeA (n = 6 mice per group). Scale bars, 40 μm (overview) and 20 μm (inset and rendering). Significance was assessed by two-tailed unpaired Student's t-test in (**b**, **d**). All data are presented as mean ± SEM. *$p < 0.05$, **$p < 0.01$, ***$p < 0.001$. See also Supplementary Data 1. Source data are provided as Source Data file.

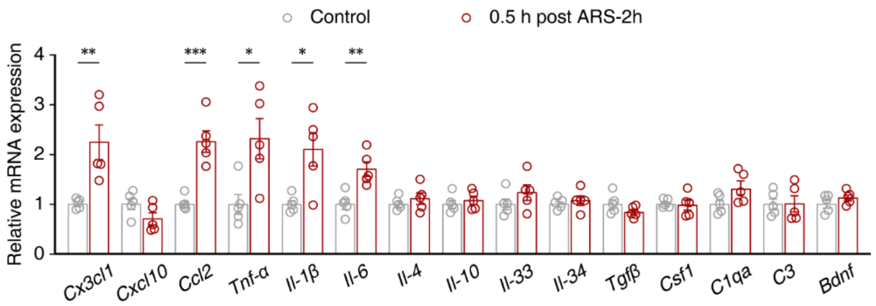

Supplementary Fig. 12: Expression of inflammatory molecules in the CeA of ARS-2h mice. qPCR analysis of mRNA levels of cytokines, chemokines, complement proteins, and growth factors in the CeA of ARS-2h mice (n = 5 mice per group; *Cx3cl1*, $t_8 = 3.540$, $p = 0.0076$; *Ccl2*, $t_8 = 5.608$, $p = 0.0005$; *Tnf-α*, $t_8 = 2.920$, $p = 0.0193$; *Il-1β*, $t_8 = 3.170$, $p = 0.0132$; *Il-6*, $t_8 = 3.892$, $p = 0.0046$). Significance was assessed by two-tailed unpaired Student's *t*-test. All data are presented as mean ± SEM. $^{*}p < 0.05$, $^{**}p < 0.01$, $^{***}p < 0.001$. See also Supplementary Data 1. Source data are provided as Source Data file.

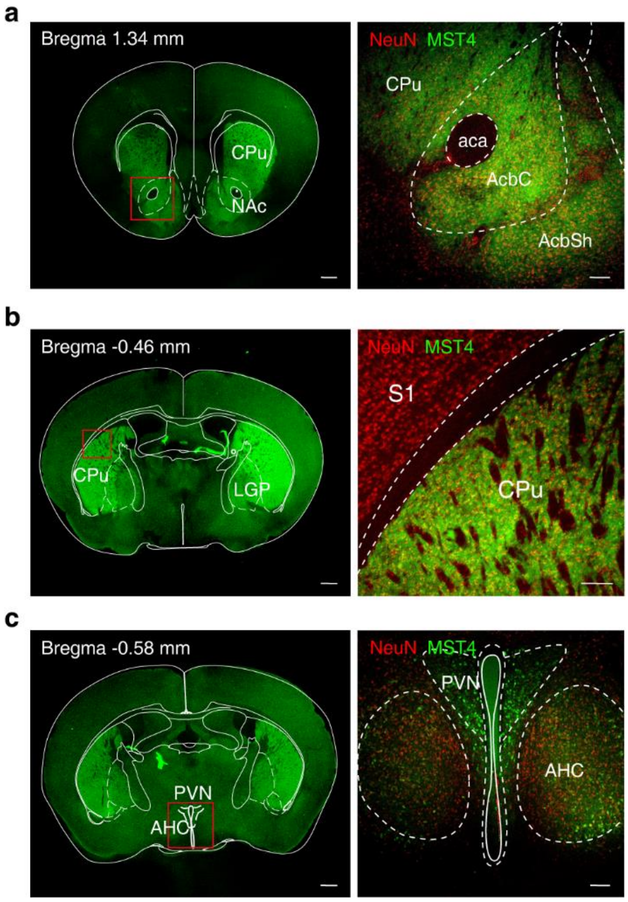

Supplementary Fig. 13: MST4 expression profile in the mouse brain.

a-**c**, Immunofluorescence staining showing the colocalization of MST4 (green) and neurons (red) in different brain regions from na ïve mice, including the accumbens nucleus (NAc, **a**), caudate putamen (CPu, **b**), paraventricular hypothalamic nucleus (PVN, **c**). Scale bars, 500 μm (left) and 100 μm (right).

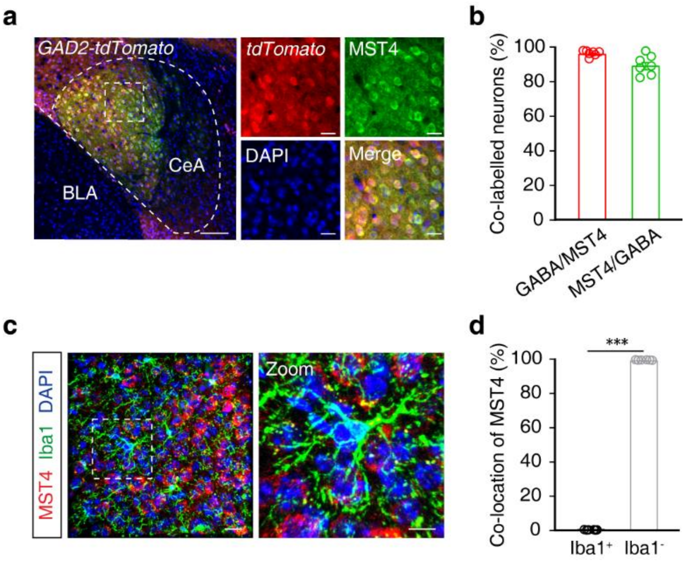

Supplementary Fig. 14: MST4 is expressed in GABA^{CeA} neurons but not in microglia.
a, Images showing co-localization of MST4-positive neurons (green) with Tdtomato^{+} neurons (red). Scale bars, 100 μm (left) and 20 μm (right). **b**, Summary data showing the percentage of MST4-positive neurons expressing GABA and GABA-positive neurons expressing MST4 in the CeA from *GAD2-tdTomato* mice (n = 7 mice per group). **c**, Representative microphotographs of double immunofluorescence staining of MST4 and Iba1^{+} microglia in the CeA. Scale bars, 20 μm (left) and 10 μm (right). **d**, Percentage data showing that Iba1^{+} microglia within the CeA were not colocalized with MST4 immunofluorescence (n = 6 mice per group; $t_{10} = 1488$, $p < 0.001$). Significance was assessed by two-tailed unpaired Student's *t*-test in (**d**). All data are presented as mean ± SEM. ***$p < 0.001$. See also Supplementary Data 1. Source data are provided as Source Data file.

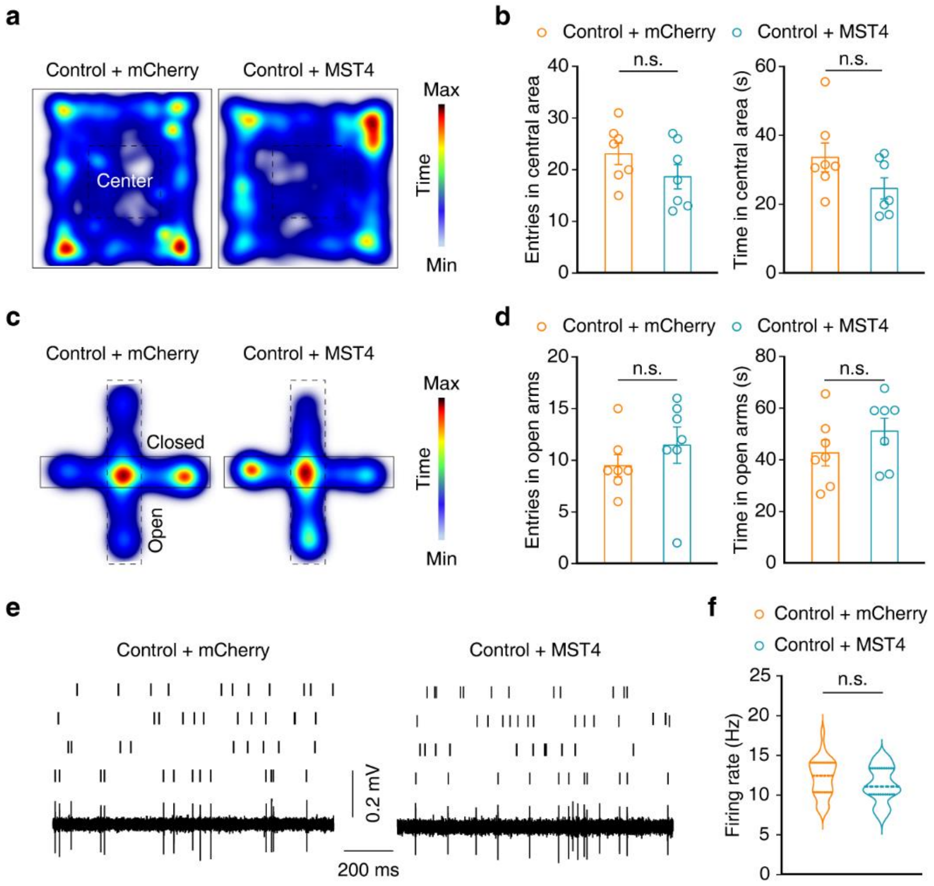

Supplementary Fig. 15: MST4 overexpression in the CeA does not affect anxiety-like behaviors in na ïve mice.

a, **b**, Representative exploration traces (**a**) and summarized data of entries and the time spent in central area (**b**) of OFT from na ïve mice infected with AAV-mCherry and AAV-MST4 (n = 7 mice per group). **c**, **d**, Representative exploration traces (**c**) and summarized data of entries and the time spent in the open arms (**d**) of EPM from na ïve mice treated with AAV-mCherry and AAV-MST4 (n = 7 mice per group). **e**, **f**, Raster plots and typical traces (**e**) and the quantitative data (**f**) of the spontaneous firings of $GABA^{CeA}$ neurons in na ïve mice infected with AAV-mCherry and AAV-MST4 (n = 30 cells from six mice per group). Significance was assessed by two-tailed unpaired Student's *t*-test in (**b**, **d**, **f**). All data are presented as mean ± SEM. n.s., not significant. See also Supplementary Data 1. Source data are provided as Source Data file.

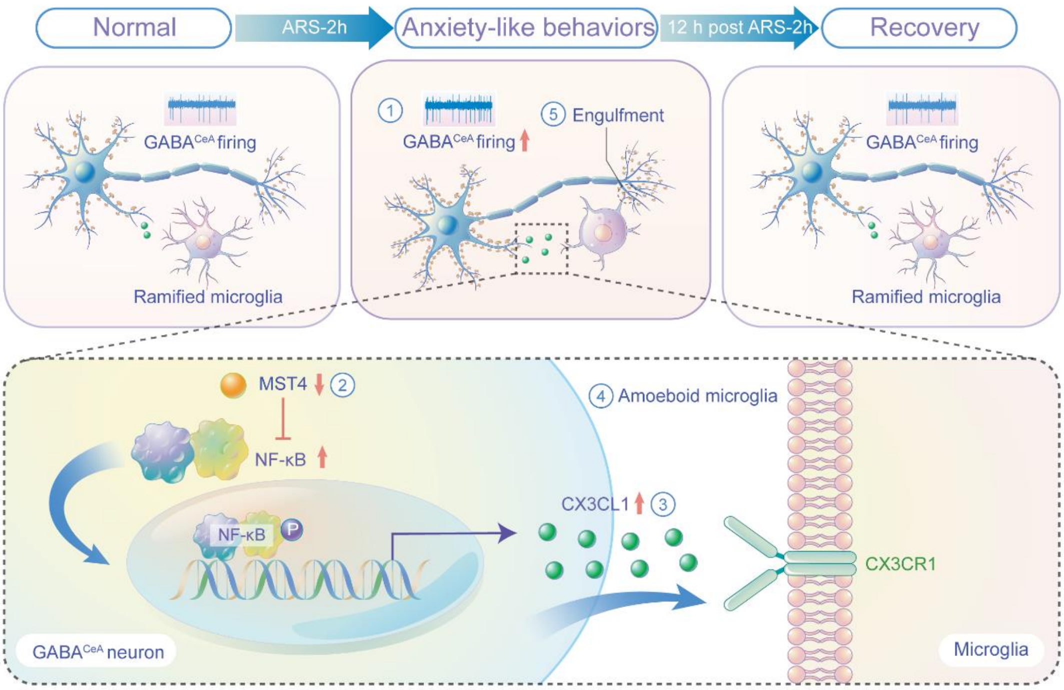

Supplementary Fig. 16: Microglial engulfment of dendritic spines promotes the extinction of acute stress-induced anxiety-like behaviors.

Anxiety-like behaviors following acute restraint are relieved within 12 hours after stress induction in male mice. Suppression of NF-κB by MST4 stimulates production of CX3CL1 by $GABA^{CeA}$ neurons, which increases under acute restraint stress and subsequently activates microglia in the CeA, promoting engulfment of dendritic spines. Microglial engulfment of dendritic spines in the CeA leads to feedback inhibition that attenuates $GABA^{CeA}$ neuronal hyperactivity, restoring them to non-stress levels and leading to extinction of anxiety-like behaviors.
