# Supplementary material for: Microglia govern the extinction of acute stress-induced anxiety-like behaviors in male mice
Source: Nat Commun. 2024 Jan 10;15:449. doi: 10.1038/s41467-024-44704-6 (PMC10781988; doi:10.1038/s41467-024-44704-6)
Supplement: Supplementary file 3 — Description of Additional Supplementary Files [file 41467_2024_44704_MOESM3_ESM.pdf]

Description of Additional Supplementary Files

Supplementary Datasets

Supplementary Data 1. Statistical analyses related to Figures 1-6 and Supplementary Figures 1-15.

Supplementary Data 2. Key resources table.

Supplementary Data 3. Primers used in this study.
