# Supplementary material for: Microglia govern the extinction of acute stress-induced anxiety-like behaviors in male mice
Source: Nat Commun. 2024 Jan 10;15:449. doi: 10.1038/s41467-024-44704-6 (PMC10781988; doi:10.1038/s41467-024-44704-6)
Supplement: Supplementary file 7 — Reporting Summary [file 41467_2024_44704_MOESM7_ESM.pdf]

nature portfolio

Corresponding author(s): Zhi Zhang, Peng Cao, Yan Jin

Last updated by author(s): Dec 13, 2023

Reporting Summary

Nature Portfolio wishes to improve the reproducibility of the work that we publish. This form provides structure for consistency and transparency in reporting. For further information on Nature Portfolio policies, see our Editorial Policies and the Editorial Policy Checklist.

Statistics

For all statistical analyses, confirm that the following items are present in the figure legend, table legend, main text, or Methods section.

n/a	Confirmed	
☐	☒	The exact sample size (*n*) for each experimental group/condition, given as a discrete number and unit of measurement
☐	☒	A statement on whether measurements were taken from distinct samples or whether the same sample was measured repeatedly
☐	☒	The statistical test(s) used AND whether they are one- or two-sided<br>*Only common tests should be described solely by name; describe more complex techniques in the Methods section.*
☒	☐	A description of all covariates tested
☐	☒	A description of any assumptions or corrections, such as tests of normality and adjustment for multiple comparisons
☐	☒	A full description of the statistical parameters including central tendency (e.g. means) or other basic estimates (e.g. regression coefficient) AND variation (e.g. standard deviation) or associated estimates of uncertainty (e.g. confidence intervals)
☐	☒	For null hypothesis testing, the test statistic (e.g. *F*, *t*, *r*) with confidence intervals, effect sizes, degrees of freedom and *P* value noted<br>*Give P values as exact values whenever suitable.*
☒	☐	For Bayesian analysis, information on the choice of priors and Markov chain Monte Carlo settings
☒	☐	For hierarchical and complex designs, identification of the appropriate level for tests and full reporting of outcomes
☒	☐	Estimates of effect sizes (e.g. Cohen's *d*, Pearson's *r*), indicating how they were calculated

Our web collection on statistics for biologists contains articles on many of the points above.

Software and code

Policy information about availability of computer code

Data collection	For slice physiological recording, the current and voltage signals were recorded with MultiClamp 700B and Clampex 10 data acquisition software (Molecular Devices, USA). For In vivo electrophysiology recording, the firing rate was recorded with Neurostudio software (Neurostudio, China). Images were captured on a Zeiss LSM880 confocal microscope (Germany) or a FV30S-SW (Olympus, Japan). Flowcytometry data were collected using CytoFLEX (Beckman Coulter, USA).
Data analysis	For slice physiological recording, the data was analyzed using Clampfit 10.7 (Molecular devices, USA) . Behavioral videos analyzed using EthoVision XT 8.5 software (Noldus). For in vivo electrophysiology recording and unsupervised clustering algorithm, the data was analyzes using Offline Sorter 4 (Plexon, USA) and Neuroexplorer 4 (Nex Technologies, USA). Flowcytometry data were analyzed using FlowJo V10. GraphPad Prism 8 (Graph Pad Software, Inc., USA) were used for the statistical analyses. Imaging data were processed and analyzed using ImageJ software (Fiji edition,NIH) or Imaris V7.

For manuscripts utilizing custom algorithms or software that are central to the research but not yet described in published literature, software must be made available to editors and reviewers. We strongly encourage code deposition in a community repository (e.g. GitHub). See the Nature Portfolio guidelines for submitting code & software for further information.

Data

Policy information about availability of data

All manuscripts must include a data availability statement. This statement should provide the following information, where applicable:

- Accession codes, unique identifiers, or web links for publicly available datasets
- A description of any restrictions on data availability
- For clinical datasets or third party data, please ensure that the statement adheres to our policy

There are no restrictions on data availability in the manuscript. The data generated in this study are provided in the Supplementary Information/Source Data file.

Research involving human participants, their data, or biological material

Policy information about studies with human participants or human data. See also policy information about sex, gender (identity/presentation), and sexual orientation and race, ethnicity and racism.

Reporting on sex and gender	*Use the terms sex (biological attribute) and gender (shaped by social and cultural circumstances) carefully in order to avoid confusing both terms. Indicate if findings apply to only one sex or gender; describe whether sex and gender were considered in study design; whether sex and/or gender was determined based on self-reporting or assigned and methods used.*<br>*Provide in the source data disaggregated sex and gender data, where this information has been collected, and if consent has been obtained for sharing of individual-level data; provide overall numbers in this Reporting Summary. Please state if this information has not been collected.*<br>*Report sex- and gender-based analyses where performed, justify reasons for lack of sex- and gender-based analysis.*
Reporting on race, ethnicity, or other socially relevant groupings	*Please specify the socially constructed or socially relevant categorization variable(s) used in your manuscript and explain why they were used. Please note that such variables should not be used as proxies for other socially constructed/relevant variables (for example, race or ethnicity should not be used as a proxy for socioeconomic status).*<br>*Provide clear definitions of the relevant terms used, how they were provided (by the participants/respondents, the researchers, or third parties), and the method(s) used to classify people into the different categories (e.g. self-report, census or administrative data, social media data, etc.)*<br>*Please provide details about how you controlled for confounding variables in your analyses.*
Population characteristics	*Describe the covariate-relevant population characteristics of the human research participants (e.g. age, genotypic information, past and current diagnosis and treatment categories). If you filled out the behavioural & social sciences study design questions and have nothing to add here, write "See above."*
Recruitment	*Describe how participants were recruited. Outline any potential self-selection bias or other biases that may be present and how these are likely to impact results.*
Ethics oversight	*Identify the organization(s) that approved the study protocol.*

Note that full information on the approval of the study protocol must also be provided in the manuscript.

Field-specific reporting

Please select the one below that is the best fit for your research. If you are not sure, read the appropriate sections before making your selection.

[x] Life sciences [ ] Behavioural & social sciences [ ] Ecological, evolutionary & environmental sciences

For a reference copy of the document with all sections, see nature.com/documents/nr-reporting-summary-flat.pdf

Life sciences study design

All studies must disclose on these points even when the disclosure is negative.

Sample size	No statistical methods were used to pre-determine sample sizes but our sample sizes are similar to those reported in previous publications (PMID: 31651582; PMID: 34233151; PMID: 36443522).
Data exclusions	For all experiments, mice with signs of infection/bleeding/unhealthy conditions after the surgeries were excluded for behavioral tests and mice with missed viral injections or implantation targets, as described by brain atlas, were not included in experimental analyses.
Replication	Behavioral experiments are replicated multiples times with independent mice, and at least two people independently analyzed time points for the behavioral events. Numbers of replicates (n) are indicated in the figure legends.
Randomization	The animals in the experiments were randomized assigned.
Blinding	All investigators were blinded to group allocation during data collection and analysis.

Reporting for specific materials, systems and methods

We require information from authors about some types of materials, experimental systems and methods used in many studies. Here, indicate whether each material, system or method listed is relevant to your study. If you are not sure if a list item applies to your research, read the appropriate section before selecting a response.

Materials & experimental systems

n/a	Involved in the study
☐	☒ Antibodies
☒	☐ Eukaryotic cell lines
☒	☐ Palaeontology and archaeology
☐	☒ Animals and other organisms
☒	☐ Clinical data
☒	☐ Dual use research of concern
☒	☐ Plants

Methods

n/a	Involved in the study
☒	☐ ChIP-seq
☐	☒ Flow cytometry
☒	☐ MRI-based neuroimaging

Antibodies

Antibodies used	For immunohistochemistry, the following antibodies were used: Rabbit anti-MST4 (AbCam, ab52491, 1:500), Goat anti-Iba1 (AbCam, ab5076, 1:500), Mouse anti-CD68 (AbCam, ab955, 1:500), Rabbit anti-GAD65/67 (AbCam, ab183999, 1:500), Mouse anti-NeuN (Millipore, MAB377, 1:500), Rabbit anti-GABA (Sigma-Aldrich, A2052, 1:500), Rabbit anti-Ki67 (Cell Signaling Technology, 12202, 1:500), Alexa fluor 488-anti-Goat secondary antibody (Invitrogen, A11055, 1:500), Alexa fluor 594-anti-Mouse secondary antibody (Invitrogen, A21203, 1:500), Alexa fluor 594-anti-Goat secondary antibody (Invitrogen, A11058, 1:500), Alexa fluor 594-anti-Rabbit secondary antibody (Invitrogen, A21207, 1:500), Alexa fluor 488-anti-Rabbit secondary antibody (Invitrogen, A21206, 1:500), Alexa fluor 647-anti-Rabbit secondary antibody (Invitrogen, A31573, 1:500).<br>For Western blot assays, the following antibodies were used: Rabbit anti-CX3CL1 (AbCam, ab25088, 1:1000), Rabbit anti-MST4 (AbCam, ab52491, 1:1000), anti-beta-actin (Absin, abs137975, 1:1000), HRP Goat Anti-Mouse lgG (H+L) (Beyotime, A0216, 1:5000), HRP Goat Anti-Rabbit lgG (H+L) (Abclonal, AS014, 1:5000).
Validation	All antibodies used in this study were obtained from commercial suppliers and were validated by the manufacturers for their specific application (immunohistochemistry, Western blot). The validation is reported on their websites.

Animals and other research organisms

Policy information about studies involving animals; ARRIVE guidelines recommended for reporting animal research, and Sex and Gender in Research

Laboratory animals	C57BL/6J mice aged 8-10 weeks purchased from Charles River used in this study;<br>Ai14 (RCL-tdT) (#007914), GAD2-Cre (#028867), and Cx3cr1-GFP (#005582) mice aged 8-10 weeks purchased from Jackson Laboratories were used in this study
Wild animals	The study did not involve wild animals.
Reporting on sex	Male mice were used in all experiments.
Field-collected samples	This study did not involve samples collected from the field.
Ethics oversight	All animal protocols were approved by the Animal Care and Use Committee of the University of Science and Technology of China.

Note that full information on the approval of the study protocol must also be provided in the manuscript.

Flow Cytometry

Plots

Confirm that:

☒ The axis labels state the marker and fluorochrome used (e.g. CD4-FITC).

☒ The axis scales are clearly visible. Include numbers along axes only for bottom left plot of group (a 'group' is an analysis of identical markers).

☒ All plots are contour plots with outliers or pseudocolor plots.

☒ A numerical value for number of cells or percentage (with statistics) is provided.

Methodology

Sample preparation	Cx3cr1-GFP mice were deeply anesthetized with pentobarbital sodium (20 mg/kg, i.p.). Subsequently, mice were perfused intracardially with 20 ml of cold Hank's Balanced Salt Solution (HBSS), followed by a rapid collection of the bilateral CeA, which was washed with cold PBS and chopped into small pieces on ice. Small tissue was mechanically homogenized using 23G needle, producing cell suspensions which were filtered through a 70 µm cell strainer. Subsequently, the samples were strained and centrifuged at 4 °C (300g, 10 min). The pellets were resuspended in 38% percoll solution and centrifuged at 4 °C (800g, 10 min). After careful removal of the supernatant, the pellets were rinsed with PBS. Finally, cells were measured using CytoFLEX (Beckman Coulter, USA) flow cytometer and data were analyzed by FlowJo V10. GFP+ cells were sorted by BD FACSAria III (BD, USA) for further experiments: investigation the gene expression of Tnf-α, Il-1β and Il-6 by Real-time PCR (Applied Biosystems, ThermoFisher, China).
Instrument	CytoFLEX
Software	FlowJo V10
Cell population abundance	At least 10^6 GFP+ cells were collected for further experiments.
Gating strategy	Gating strategy, with representative gating, is provided in Supplementary Fig. 5.<br>Briefly, FSC-A/ SSC-A were used to identify viable cells. FSC-A/ FSC-H gating was used to identify singlet cells. FITC-A/ SSC-H gating was used to identify GFP+ cells.

☒ Tick this box to confirm that a figure exemplifying the gating strategy is provided in the Supplementary Information.
